# Whole brain proton irradiation in adult Sprague Dawley rats produces dose dependent and non-dependent cognitive, behavioral, and dopaminergic effects

**DOI:** 10.1038/s41598-020-78128-1

**Published:** 2020-12-09

**Authors:** Michael T. Williams, Chiho Sugimoto, Samantha L. Regan, Emily M. Pitzer, Adam L. Fritz, Anthony E. Mascia, Mathieu Sertorio, Ralph E. Vatner, John P. Perentesis, Charles V. Vorhees

**Affiliations:** 1 Division of Neurology (MLC 7044), Cincinnati Children’s Research Foundation, 3333 Burnet Ave., Cincinnati, OH 45229-3039 USA; 2 Department of Pediatrics, University of Cincinnati College of Medicine, Cincinnati, OH 45229 USA; 3 Department of Radiation Oncology, University of Cincinnati College of Medicine, Cincinnati, OH 45267 USA; 4 Division of Oncology, Cincinnati Children’s Research Foundation, Cincinnati, OH 45229 USA; 5 Cincinnati Children’s/University of Cincinnati Proton Therapy and Research Center, Cincinnati, OH 45229 USA

**Keywords:** CNS cancer, Hippocampus, Long-term memory, Spatial memory, Neuroscience, Learning and memory

## Abstract

Proton radiotherapy causes less off-target effects than X-rays but is not without effect. To reduce adverse effects of proton radiotherapy, a model of cognitive deficits from conventional proton exposure is needed. We developed a model emphasizing multiple cognitive outcomes. Adult male rats (10/group) received a single dose of 0, 11, 14, 17, or 20 Gy irradiation (the 20 Gy group was not used because 50% died). Rats were tested once/week for 5 weeks post-irradiation for activity, coordination, and startle. Cognitive assessment began 6-weeks post-irradiation with novel object recognition (NOR), egocentric learning, allocentric learning, reference memory, and proximal cue learning. Proton exposure had the largest effect on activity and prepulse inhibition of startle 1-week post-irradiation that dissipated each week. 6-weeks post-irradiation, there were no effects on NOR, however proton exposure impaired egocentric (Cincinnati water maze) and allocentric learning and caused reference memory deficits (Morris water maze), but did not affect proximal cue learning or swimming performance. Proton groups also had reduced striatal levels of the dopamine transporter, tyrosine hydroxylase, and the dopamine receptor D1, effects consistent with egocentric learning deficits. This new model will facilitate investigations of different proton dose rates and drugs to ameliorate the cognitive sequelae of proton radiotherapy.

## Introduction

The use of therapeutic ionizing radiation has prolonged the life of cancer patients, but it can also impair neurocognitive function^1–4^. Cranial irradiation, in particular, is an essential palliative and curative treatment for many brain tumors and can prevent against development of brain metastases for some carcinomas and leukemias. Proton radiation therapy has been used for cancer treatment since the 1960s, but until recently its use was limited by the number of available facilities^5^. Proton radiotherapy can decrease toxicity by reducing radiation exposure of healthy brain tissue, while delivering equivalent anti-tumor efficacy compared with X-ray radiotherapy^6,7^. In that regard, a recent pivotal study in children with medulloblastoma identified superior intellectual outcomes in patients treated with proton compared with photon radiotherapy^8^. Furthermore, the use of proton therapy versus photons for pediatric and adult CNS tumors lowers the incidence of second primary cancers^9^. There are a number of preclinical studies that examined proton exposure in the rat and its effects on cognition, although most of these were at lower dose levels^10–13^. Preclinical studies examining proton exposure at therapeutic doses have focused on tissue-related effects and not functional outcomes^14–17^. Other types of radiation at therapeutic doses also affect cognition^18–23^.

Given the heterogeneous composition of the brain, whole brain irradiation may have differential effects in different brain regions. For example, in a retrospective study using magnetic resonance imaging of brain tumor patients who had whole brain irradiation, the entorhinal and inferior parietal cortices had more cortical thinning after radiation treatment, but there was no thinning in the primary visual and somatosensory cortices^24^. Cortical thinning was dose related, however, this study only had 56 patients and only the cortex was examined. After proton radiation doses of 22–101 Gy delivered to the whole body of rats, differential neurotoxicity was found with the cortex > striatum, hypothalamus, hippocampus > substantia nigra and pons, although this study had few rats at each dose and measured only acute neurotoxicity 4 days after irradiation^17^. Taken together these data suggest the need to investigate a range of CNS functions. Therefore, the effects of whole brain proton irradiation on healthy tissue were investigated in rats using a comprehensive approach that included a battery of structure/function behavioral methods. Given that the striatum and hippocampus were identified as sensitive regions following proton irradiation, the Cincinnati water maze (CWM) was used since it is largely a striatally mediated task^25^ and the Morris water maze (MWM) since it is a hippocampally mediated task^26^. Furthermore, novel object recognition (NOR) is dependent upon the hippocampus and other nearby structures^27^. Locomotor behavior is primarily dependent upon the striatum and substantia nigra^28^ whereas acoustic and tactile startle both project to the caudal pontine reticular nucleus^29^. Five doses of proton irradiation, 0, 11, 14, 17, and 20 Gy at a clinical dose rate of 1 Gy/s were used. Rats were tested for acute changes and long-term cognitive effects. Cognitive deficits were predicted based on clinical and animal data and therefore one-tailed tests were used. For the first 5 weeks after irradiation, rats were tested in an open-field for exploratory and habituation behavior, acoustic and tactile startle responses (ASR and TSR, respectively) for sensory integrity to external stimuli, rotorod for motor coordination, and bright light prepulse inhibition of ASR/TSR for attention/sensorimotor gating. Differences in behavior after treatment compared with sham controls on these tests provide data on the vulnerability of different brain regions to proton irradiation. After testing, brain tissues were analyzed by western blot for neurotransmitter systems important in egocentric and allocentric learning and memory. In particular the dopamine system was examined since it is important in locomotion and CWM^25,28^, NMDA subunits were assessed since they are involved in spatial learning and memory^30^, Iba1 and GFAP as markers of reactive gliosis^31^, and Ki67 as a marker for possible changes in cell proliferation^32^.

## Results

### Mortality

There were 10 rats in each group with the exception of the 20 Gy group that had 11 rats. For the 11 rats that were irradiated in the 20 Gy group, 6 rats died > 4 days and < 2 weeks after irradiation; no autopsy was performed. One rat died within 5 days, 1 within 11 days, 2 within 12 days, and 1 within 14 days following irradiation. No rats in the 17 Gy or 11 Gy groups died, and 2 rats died in the 14 Gy group more than 2 months after irradiation from malocclusion, not radiation. Because of the high mortality in the 20 Gy group, they were not included in the data analyses.

### Body weights

**Figure 1:**
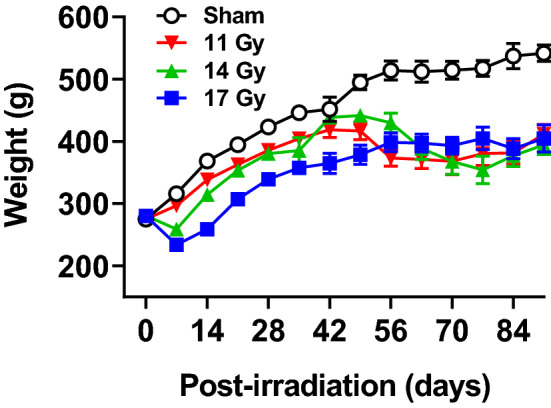
Body weights (mean ± SEM) of proton irradiated and control rats. There were no differences in weight at the time of irradiation. Regardless of dose, irradiated rats had decreased body weights (*p* < 0.0001). The 11 Gy rats had decreased weights at 5 weeks, no difference at 6 weeks, and decreased weights the rest of the testing period compared with controls.. The 14 Gy and 17 Gy rats had decreased weights at week 1 until the end of testing with the exception of week 6 in the 14 Gy rats when there was no difference compared with controls. n = 10/group for sham controls, 11 Gy, and 17 Gy and 8–10 for 14 Gy.

All proton exposed rats had decreased body weights compared with controls [group: F(3, 42) = 23.07, *p* < 0.0001], and all rats gained weight [week: F(13, 378) = 40.03, *p* < 0.0001] (Fig. 1). However, there was an interaction of group × week [F(39, 403) = 5.14, *p* < 0.0001]. There were no differences in weight on the day of irradiation. Beginning 1 week later, the 14 and 17 Gy groups weighed less than controls and remained lighter for the rest of the experiment except on week 6 when the 14 Gy group did not differ from control. The 11 Gy group had weight reductions beginning at 5 weeks, no difference at 6 weeks, and reductions thereafter for the remainder of the experiment compared with controls.

### Locomotor activity

**Figure 2:**
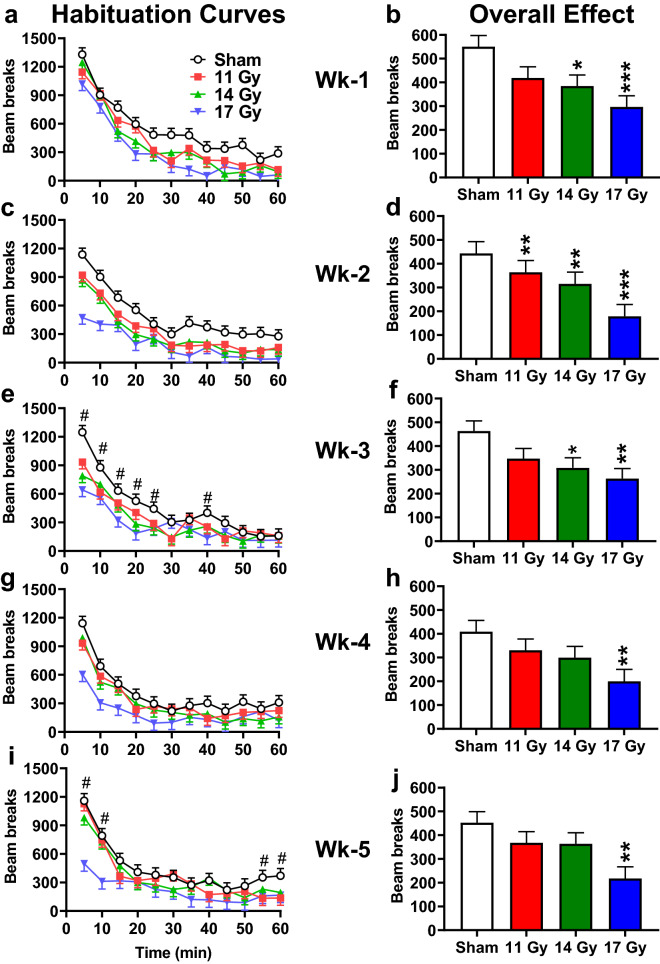
Weekly locomotor activity in proton irradiated and control rats. (**a**) Week-1 habituation curve. (**b**) Week-1 overall effects. (**c**) Week-2 habituation curve. (**d**) Week-2 overall effects. (**e**) Week-3 habituation curve. (**f**) Week-3 overall effects. (**g**) Week-4 habituation curve. (**h**) Week-4 overall effects. (**i**) Week-5 habituation curve. (**j**) Week-5 overall effects. For week 3 and 5 there were interactions. # week-3 (**e**) 11 Gy rats had reduced locomotion at 5-min, 14 Gy at 5 and 20 min, and 17 Gy at 5–25 and 40 min compared with controls. # week-5 (**i**) 11 Gy rats had reduced locomotion at 55 and 60 min and 17 Gy at 5–10 min compared with controls. *p < 0.05, **p < 0.01, ***p < 0.001 compared with controls. N = 10/group for all time points except at Week-4 for the 17 Gy, n = 9.

Spontaneous locomotor activity was assessed weekly for 5 weeks following irradiation as a measure of exploration and habituation to an open environment. At week-1 post-irradiation (Fig. 2a), total ambulation was significantly reduced in the 14 and 17 Gy groups (*p* < 0.02 and 0.0007) with the 11 Gy group not differing significantly (*p* < 0.06) compared with controls [F(3, 33.9) = 4.92, *p* < 0.007] (Fig. 2b). All rats explored the open-field initially then gradually habituated; a pattern observed on all weeks [Week 1: F(11, 335) = 70.55, *p* < 0.0001; Week 2: F(11, 321) = 34.27, *p* < 0.0001; Week 3: F(11, 323) = 30.35, *p* < 0.0001; Week 4: F(11, 298) = 35.14, *p* < 0.0001; Week 5: F(11, 183) = 26.34, *p* < 0.0001; (Fig. 2a,c,e,g,i)]. The interaction of group × time was not significant on weeks 1, 2, or 4.

For weeks 2–5, there were main effects of group: Week 2 [F(3, 33.6) = 9.62, *p* < 0.0001] (Fig. 2d); Week 3 [F(3, 37.3) = 3.74, *p* < 0.02] (Fig. 2f); Week 4 [F(3, 32.3) = 3.09, *p* < 0.05] (Fig. 2h); Week 5 [F(3, 23.3) = 4.05, *p* < 0.02] (Fig. 2j). At week 2, all irradiated groups had reduced ambulation (11 Gy, *p* < 0.01, 14 Gy *p* < 0.002, and 17 Gy *p* < 0.0001), whereas at week 3 only the 14 and 17 Gy groups (*p* < 0.02 and 0.003) had reduced ambulation compared with controls. On week 3, the group × time interaction was also significant [F(33, 351) = 1.67, *p* < 0.02]. Compared with controls, the 11 Gy group had reduced ambulation at 5 min, the 14 Gy group at 5 and 20 min, and the 17 Gy group at 5–25 and 40 min. At 4 and 5 weeks only the 17 Gy group (*p* < 0.006 and 0.003) had reduced ambulation compared with controls. On week 5 the group × time interaction was significant [F(33, 250) = 1.69, *p* < 0.02]. Compared with controls, the 17 Gy group had reduced ambulation at 5–10 min and the 11 Gy group at 55 and 60 min, no differences were observed for the 14 Gy group.

### Acoustic and tactile startle

**Figure 3:**
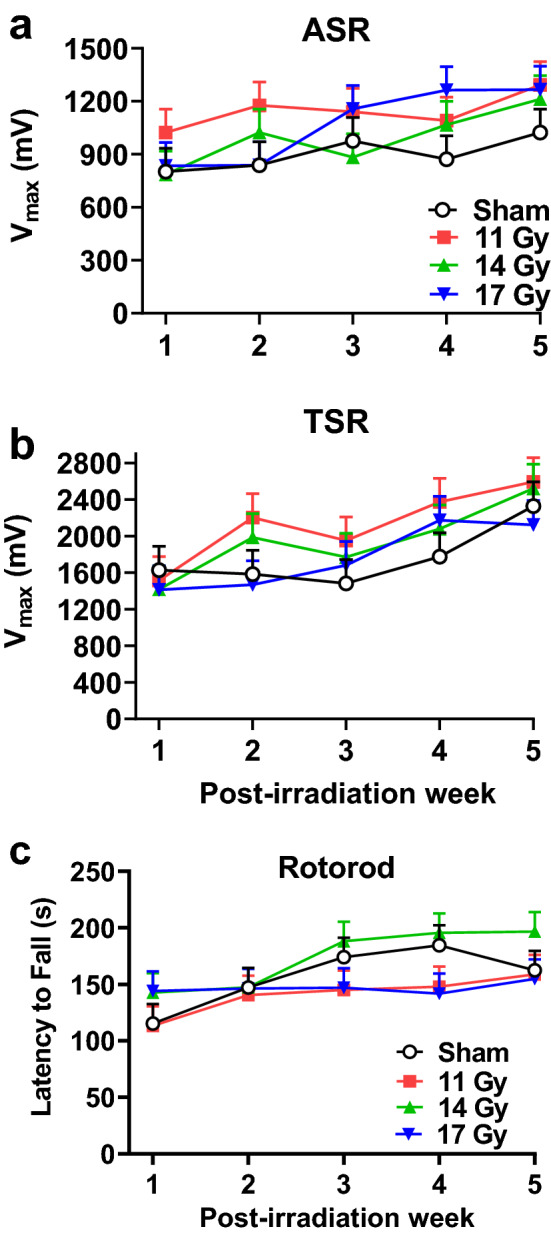
(**a**) ASR each week after proton exposure. (**b**) TSR each week after proton exposure. No differences were found for ASR or TSR. N = 10/group each week. (**c**) Weekly average latency to fall in the rotorod test. No differences were found. N = 10/group each week with the exception of week-4 when there were only 9 for the control, 11, and, 17 Gy groups.

Acoustic and tactile startle were also tested weekly after locomotor testing as a measure of reflexive responses to a loud mixed frequency signal and an air-puff. For acoustic startle, there were no differences between the irradiated groups and controls (Fig. 3a). For all groups there was an increase in peak response on weeks following week 1 (F(4, 94.9) = 5.58, *p* < 0.0001) but no interaction with week. Similarly, for tactile startle (Fig. 3b), there were no differences between the irradiated groups and controls, nor was there an interaction with week. There was a time/age increase in response for all groups on weeks 2–5 compared with week 1 (F(4, 123) = 7.46, *p* < 0.0001).

### Rotorod

To assess coordination, a rotorod was used. There were no differences between the irradiated groups and the controls on rotorod for latency to fall (Fig. 3c). Rats improved performance over weeks (F(4, 104) = 4.22, *p* < 0.004); there was no interaction of group × week.

### Bright light prepulse inhibition of startle (PPI)

**Figure 4:**
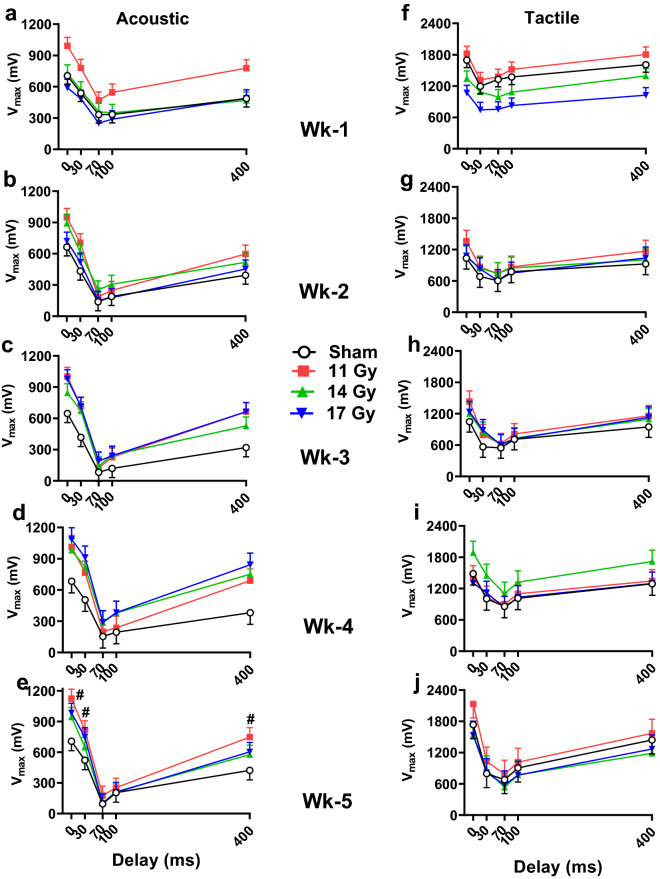
Weekly light PPI of ASR (intervals of 30, 70, 100, and 400 ms) (**a–e**) and light PPI of TSR (**f-j**) after proton exposure. The 11 Gy group had increased ASR at weeks 1, 3, and 5 (**a,c,e**: *p* < 0.05) and the 17 Gy group had increased ASR at weeks 3 and 4 (**c,d**: *p* < 0.05), regardless of prepulse compared with the controls. At week 5 for ASR there was an interaction, #*p* < 0.05 compared with control. The 11 and 17 Gy groups had increased ASR when the response was unmodified. The 11 Gy rats had increased ASR at 30 and 400 ms. For TSR the only effect was at week 1 (**f**) when the 17 Gy rats had smaller responses compared with controls (main effects have no significance indicators on the figure). N = 10/group/time point.

Light PPI was examined each week after rotorod as a test of sensorimotor gating. When there was an effect on ASR, the irradiated groups had increased startle amplitude compared with controls regardless of prepulse by Dunnett’s (Fig. 4a–e). Specifically, the increased startle occurred at week 1 (Fig. 4a), week 3 (Fig. 4c), and week 5 (Fig. 4e) for the 11 Gy group (*p* < 0.03, 0.04, and 0.05, respectively), with no differences at 2 weeks for any of the groups (Fig. 4b), and increased responses at weeks 3 and 4 (Fig. 4c,d) in the 17 Gy group (*p* < 0.04). For week 5 (Fig. 4e), there was an interaction of group × prepulse [F(12, 144) = 1.98, *p* < 0.04] with the 11 and 17 Gy groups having increased responses on no prepulse trials. Furthermore, the 11 Gy group had greater responses at the 30 and 400 ms intervals compared with controls. On each week, regardless of group, the rats showed the typical differences in response to the prepulse intervals (Week 1: F(4, 74.8) = 64.30; Week 2: (4, 92.3) = 135.26; Week 3: F(4, 144) = 151.25; Week 4: F(4, 81.3) = 119.28; Week 5: F(4, 144) = 182.18; all *p* < 0.0001). Other than week 5, there were no other interactions for ASR.

For TSR with light PPI (Fig. 4f–j) there was only an effect at week 1 (Fig. 4f) with the 17 Gy group responding less than controls regardless of prepulse (*p* < 0.006) with no differences in the 11 or 14 Gy groups compared with controls. On each week, all groups had expected differences in response to the prepulse intervals (Week 1: F(4, 83.9) = 42.96; Week 2: F(4, 144) = 32.38; Week 3: F(4, 144) = 47.03: Week 4: F(4, 77.4) = 33.99; Week 5: F(4, 53.9) = 59.83; all *p* < 0.0001). There were no other differences on weeks 2–5 (Fig. 4g–j) and no interactions.

### Novel object recognition

Cognitive tests began 6 weeks after irradiation. The first was novel object recognition. There were no differences between irradiated and control groups for percent time spent with the novel object. The means ± SEM were as follows: control, 34.9 ± 4.6%; 11 Gy, 35.4 ± 4.6%; 14 Gy, 34.7 ± 4.9%; and 17 Gy, 39.2 ± 4.6%.

### Straight channel swim

**Figure 5:**
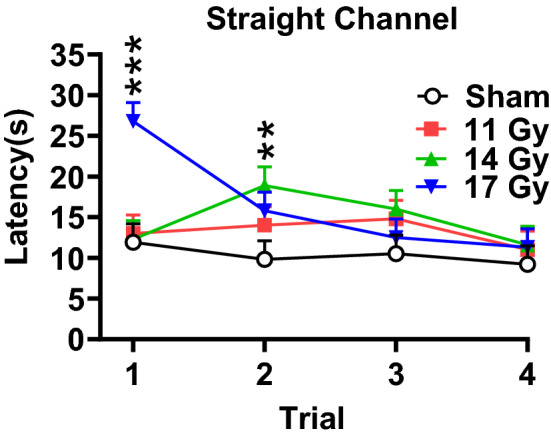
Straight channel swim latencies by trial to locate the hidden platform of proton irradiated and control rats. **p < 0.01 14 Gy versus controls, ***p < 0.001 17 Gy versus controls. N = 10/group.

Prior to water mazes, rats received swimming experience in a straight channel to determine motivation to escape and let them discover how to escape. There was a main effect of group [F(3, 37.6) = 4.21, *p* < 0.02]. The 17 Gy (*p* < 0.002) and the 14 Gy group (*p* < 0.03) swam slower than the control group overall, but the effect was trial-specific; the 11 Gy group did not differ from controls (Fig. 5). The interaction of group × trial [F(9, 90.9) = 3.12, *p* < 0.003] showed that on trial 1, the 17 Gy group reached the goal slower than controls (*p* < 0.001), whereas on trial 2 the 14 Gy group took longer than controls (*p* < 0.01). There were no differences between irradiated groups and the control group on trials 3 and 4, such that all groups were equivalent when entering the CWM the next day. All groups improved performance across trials [F(3, 80.3) = 3.84, *p* < 0.02].

### Cincinnati water maze

**Figure 6:**
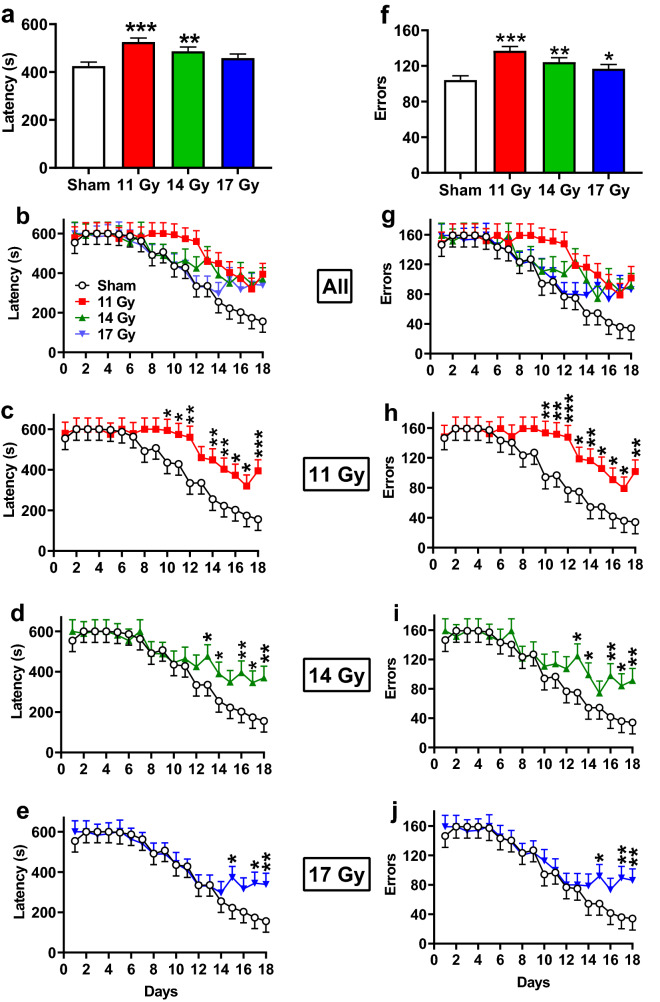
Cincinnati water maze performance in proton irradiated and control rats. (**a**) Overall effect on latency to the platform. (**b**) Learning curve for latency with all groups. (**c**) Learning curve for latency with 11 Gy and control. (**d**) Learning curve for latency with 14 Gy and control. (**e**) Learning curve for latency with 17 Gy and control. (**f**) Overall effect on maximum errors. (**g**) Learning curve for maximum errors with all groups. (**h**) Learning curve for maximum errors with 11 Gy and control. (**i**) Learning curve for maximum errors with 14 Gy and control. (**j**) Learning curve for maximum errors with 17 Gy and control. *p < 0.05, **p < 0.01, ***p < 0.001 compared with controls. N = 10/control, 11 Gy, and 17 Gy and n = 9 for the 14 Gy.

The CWM is a 10-unit T-maze that tests egocentric striatally mediated learning and memory by testing rats in the dark to remove visual cues. The 11 and 14 Gy groups (*p* < 0.0001 and *p* < 0.006, respectively) took longer to locate the escape platform compared with controls, while there was no difference in the 17 Gy group (*p* < 0.08) (Fig. 6a). All groups showed improvement in performance over the 18 days of testing (Fig. 6b, F(17, 465) = 10.25, *p* < 0.0001), and there was no interaction of group × day. Because CWM data are censured on early trials, we conducted slice effect ANOVAs by day. Latencies were increased for the 11 Gy group on days 10–18 (Fig. 6c), for the 14 Gy group on days 13–18 but not day 15 (*p* < 0.06) (Fig. 6d), and for the 17 Gy group on days 15, 17, 18 but not day 16 (*p* < 0.07) (Fig. 6e) compared with the control group.

A similar pattern was found for errors, with the exception that all irradiated groups made more errors than the control group (Fig. 6f: 11 Gy, *p* < 0.0001, 14 Gy, *p* < 0.003, 17 Gy, *p* < 0.04). Regardless of group, rats reduced errors over days (Fig. 6g, F(17, 473) = 10.25, *p* < 0.0001); there was no interaction of group × day. As for latencies, slice effect ANOVAs for errors on each day were examined. Increased errors were observed for the 11 Gy group on days 10–18 (Fig. 6h), for the 14 Gy group from days 13, 14, 16–18 (Fig. 6i), and for the 17 Gy groups on days 15, 17, 18 (Fig. 6j).

### Morris water maze

**Figure 7:**
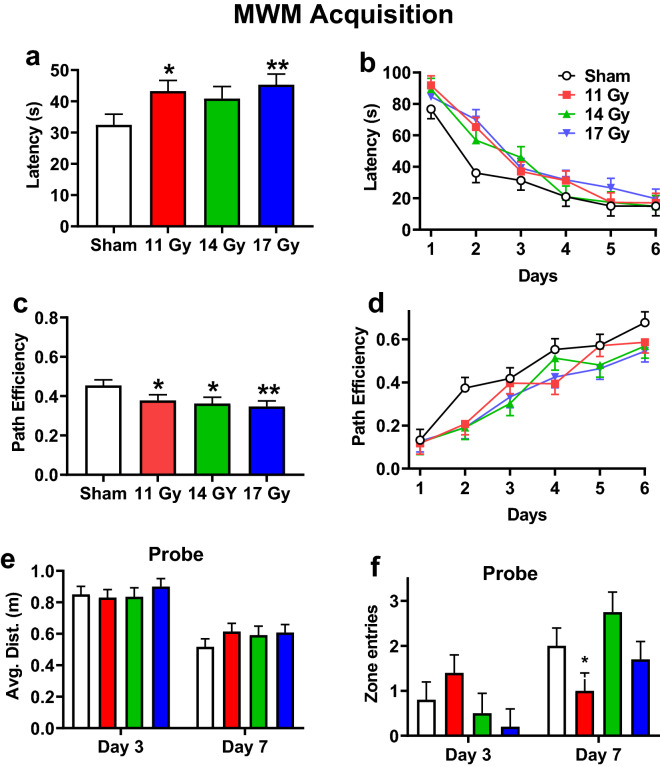
Acquisition phase Morris water maze performance in proton irradiated and control rats. (**a**) Overall effect on latency to the platform. (**b**) Learning curve for latency with all groups. (**c**) Overall effect on path efficiency. (**d**) Learning curve for path efficiency. **e.** Average distance from the former platform site on day-3 and day-7 probe trials. (**f**) Number of entries into the former platform site on day-3 and -7 probe trials. There was an interaction for entries with the 11 Gy rats having fewer entries than the controls. *p < 0.05, **p < 0.01 compared with controls. N = 10/control, 11 Gy, and 17 Gy and n = 8 for the 14 Gy.

The Morris water maze tests allocentric hippocampally mediated learning and memory, since rats use distal cues to navigate to the platform. There were 4 phases: acquisition, reversal, shift, and cued-random. For acquisition, the 11 and 17 Gy groups (*p* < 0.02 and *p* < 0.006, respectively) had longer latencies but not the 14 Gy group (*p* < 0.06) (Fig. 7a) compared with controls. Rats found the platform faster over days [F(5, 123) = 56.87, *p* < 0.0001]; there was no interaction of group × day (Fig. 7b). All irradiated groups were less efficient at reaching the platform compared with controls (Fig. 7c: 11 Gy, *p* < 0.04; 14 Gy, *p* < 0.02; 17 Gy, *p* < 0.006). Regardless of group, efficiency increased over days [F(5, 123) = 36.46, *p* < 0.0001], and there was no interaction of group × day (Fig. 7d). The swim speed of rats was comparable across groups. The mean ± SEM for swim speeds were: control, 22.3 ± 0.7 cm/s; 11 Gy, 22.1 ± 0.7 cm/s; 14 Gy, 23.7 ± 0.8 cm/s; and 17 Gy, 22.8 ± 0.7 cm/s.

For the probe trials after acquisition, there were no differences for average distance between irradiated groups and controls (Fig. 7e). Regardless of group, rats had shorter average distances on the last day compared with day-3 probe trials [F(1, 34) = 76.41, *p* < 0.0001]. There was no interaction. For entries in the former platform site (Fig. 7f), there was no difference between irradiated groups and controls. Regardless of group, rats had more entries on the last day compared with day-3 [F(1, 34) = 18.82, *p* < 0.0001]. There was an interaction of group × day [F(3, 34) = 4.48, *p* < 0.01]. There were no differences on day-3, however on the day-7 probe the 11 Gy group had fewer entries than controls (Fig. 7f).

**Figure 8:**
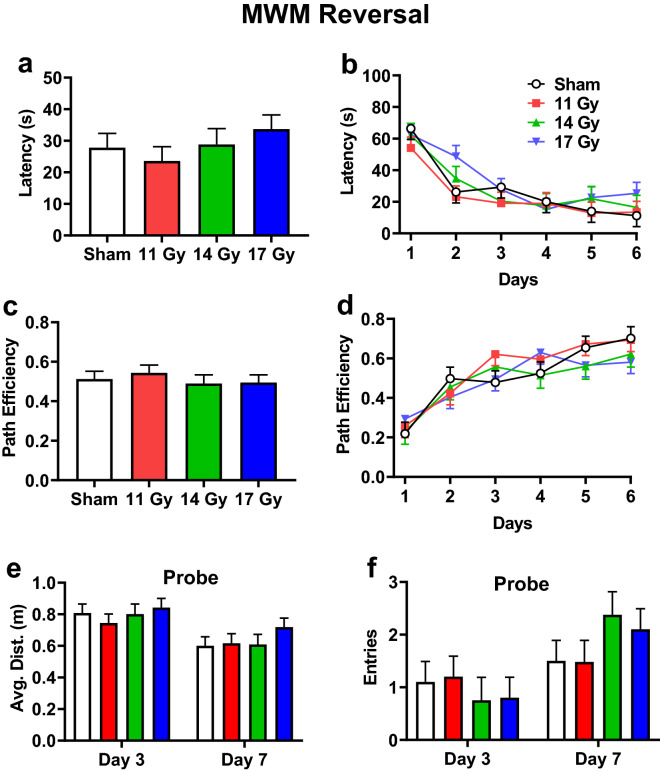
Reversal phase Morris water maze performance in proton irradiated and control rats. (**a**) Overall effect on latency to the platform. (**b**) Learning curve for latency with all groups. (**c**) Overall effect on path efficiency. (**d**) Learning curve for path efficiency. **e.** Average distance from the former platform site on day-3 and -7 probe trials. (**f**) Number of entries into the former platform site on day-3 and day-7 probe trials. No group differences were found. N = 10/control, 11 Gy, and 17 Gy and n = 8 for the 14 Gy.

For reversal, there were no differences in latency (Fig. 8a); regardless of group, rats found the platform faster over days [Fig. 8b: F(5, 120) = 24.20, *p* < 0.0001]. There was no interaction. Similarly, there were no differences in path efficiency (Fig. 8c), and rats became more efficient over days [Fig. 8d: F(5, 138) = 26.21, *p* < 0.0001]. There was no interaction for path efficiency. There were no differences in swim speed. The mean ± SEM for swim speeds were as follows: control, 22.0 ± 0.8 cm/s; 11 Gy, 20.7 ± 0.8 cm/s; 14 Gy, 20.8 ± 0.9 cm/s; and 17 Gy, 21.8 ± 0.8 cm/s. For the probe trials, there were no differences in average distance to the platform (Fig. 8e) or entries (Fig. 8f) between the irradiated and control groups. The average distance decreased [F(1, 33.9) = 20.84, *p* < 0.0001] and the number of entries increased between day-3 and day-7 [F(1, 33.7) = 16.48, *p* < 0.0003]. There was no interaction for average distance or entries.

**Figure 9:**
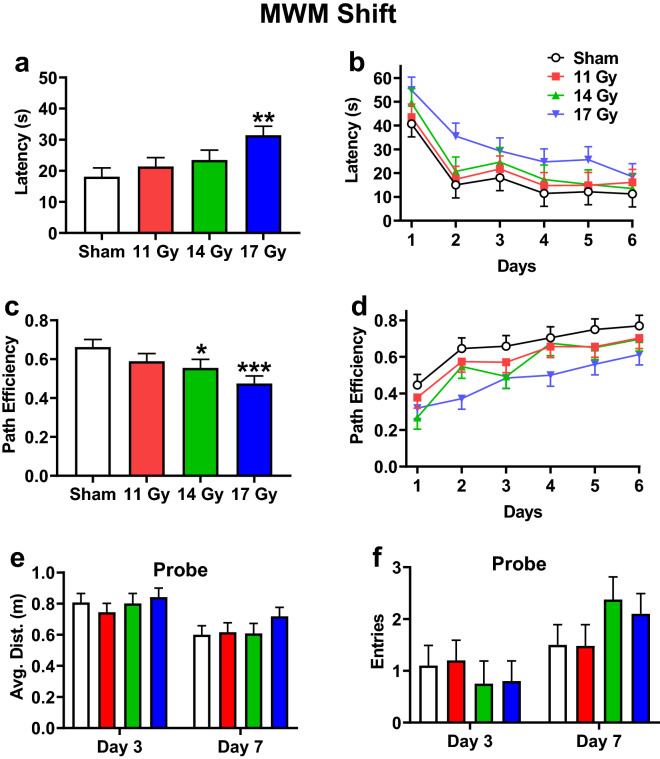
Shift phase Morris water maze performance in proton irradiated and control rats. (**a**) Overall effect on latency to the platform. (**b**) Learning curve for latency with all groups. (**c**) Overall effect on path efficiency. (**d**) Learning curve for path efficiency. (**e**) Average distance from the former platform site on day-3 and -7 probe trials. (**f**) Number of entries into the former platform site on day-3 and day-7 probe trials. There was an interaction for entries with the 11 Gy rats having fewer entries than the controls. *p < 0.05, **p < 0.01, ***p < 0.001 compared with controls. N = 10/control, 11 Gy, and 17 Gy and n = 8 for the 14 Gy.

For shift, the 17 Gy group had increased latencies compared with the control group (*p* < 0.002) with no differences in the 11 or 14 Gy groups (Fig. 9a). Regardless of group, rats had decreased latencies over days [Fig. 9b: F(5, 138) = 19.50, *p* < 0.0001], and there was no interaction. The 14 and 17 Gy groups (*p* < 0.04 and *p* < 0.001, respectively) were less efficient in reaching the platform than the control group, with no difference in the 11 Gy group (Fig. 9c). Efficiencies increased across days in all groups [F(5, 127) = 25.36, *p* < 0.0001] and there were no interactions (Fig. 9d). There were no differences in swim speed. The mean ± SEM for speed were as follows: control, 20.5 ± 0.9 cm/s; 11 Gy, 20.3 ± 0.9 cm/s; 14 Gy, 20.2 ± 1.0 cm/s; and 17 Gy, 22.3 ± 0.9 cm/s. For the probe trials, there was no difference between irradiated groups and controls for average distance (Fig. 9e) or entries (Fig. 9f). All groups had a decrease in average distance from day-3 to day-7 [F(1, 33.9) = 20.84, *p* < 0.0001] and more entries [F(1, 33.7) = 16.48, *p* < 0.0003]. There were no interactions for either average distance or entries.

**Figure 10:**
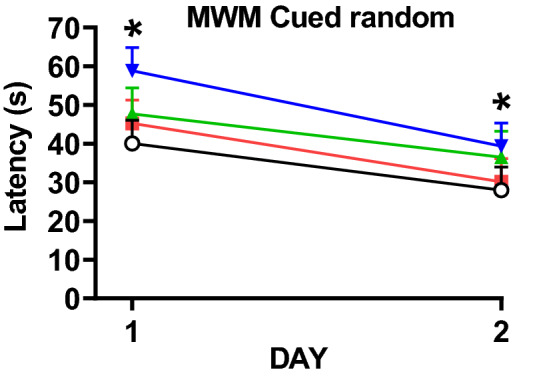
Cued-random phase Morris water maze performance in proton irradiated and control rats. *p < 0.05, 17 Gy compared with controls. N = 10/control, 11 Gy, and 17 Gy and n = 8 for the 14 Gy.

For the cued-random phase, curtains were drawn around the tank to obscure distal cues and a proximal cue was mounted on the platform. The 17 Gy group had longer latencies to reach the platform than the control group (*p* < 0.02), while there were no differences in the 11 or 14 Gy groups compared with controls (Fig. 10). All groups had a reduction in latency over days [F(1, 34) = 14.96, *p* < 0.0005], and there was no interaction of exposure × day.

### Western blots

**Figure 11:**
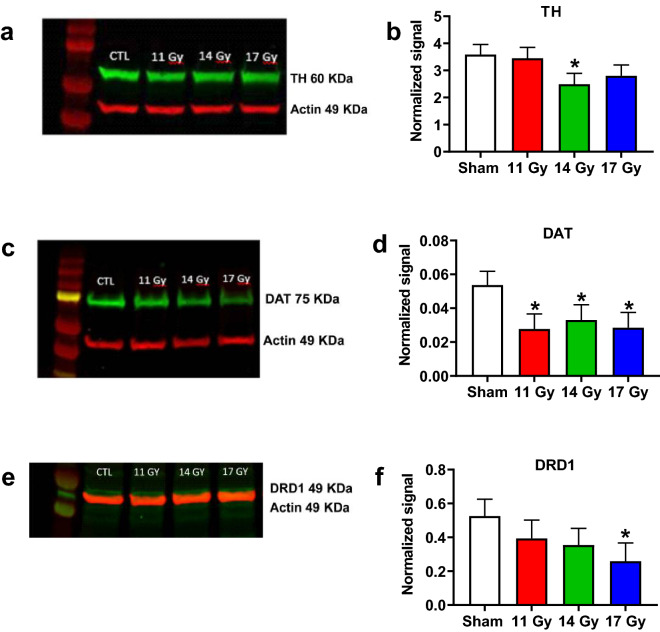
Western blot data for TH (**a**,**b**), DAT (**c**,**d**), and DRD1 (**e**,**f**) for proton irradiated and control rats. Full-length blots/gels are presented in Supplementary Materials Figure S1a–c. *p < 0.05 compared with controls. N = 5–6/group.

At the end of behavioral testing brains were dissected and western blots were run for dopamine, glutamate, astrocytic, and microglial biomarkers in the neostriatum and hippocampus, including tyrosine hydroxylase (TH), dopamine transporter (DAT), and dopamine receptor D1 (DRD1). For neostriatum, TH was decreased in the 14 Gy group (*p* < 0.04) compared with controls (Fig. 11a,b). There was no difference in the 11 and 17 Gy groups. DAT was decreased in the neostriatum of the 11 Gy (*p* < 0.03), 14 Gy (*p* = 0.05) and 17 Gy (*p* < 0.03) groups, compared with controls (Fig. 11c,d). DRD1 was decreased in the 17 Gy group (*p* < 0.05) compared with controls with no differences in the 11 and 14 Gy groups (Fig. 11e,f). No differences between irradiated groups and controls in the neostriatum were found for IBA1, GFAP, or Ki67 (Table 1). In the hippocampus, no differences were found for any of the biomarkers (Table 2).

**Table 1:** Striatal markers.

Marker	Treatment	N	LS means (normalized to actin)	SEM
GFAP	Control	6	1.22	0.17
	11 Gy	6	0.99	0.17
	14 Gy	6	1.01	0.17
	17 Gy	6	0.89	0.17
Iba1	Control	5	0.47	0.10
	11 Gy	5	0.59	0.10
	14 Gy	5	0.64	0.10
	17 Gy	5	0.55	0.10
Ki67	Control	6	0.15	0.02
	11 Gy	5	0.14	0.02
	14 Gy	5	0.16	0.02
	17 Gy	5	0.13	0.02

**Table 2:** Hippocampal markers.

Marker	Treatment	N	LS means (normalized to actin)	SEM
GFAP	Control	6	1.20	0.13
	11 Gy	6	1.24	0.13
	14 Gy	6	1.13	0.13
	17 Gy	6	1.04	0.13
Iba1	Control	6	0.17	0.02
	11 Gy	6	0.21	0.02
	14 Gy	6	0.18	0.02
	17 Gy	5	0.19	0.03
Ki67	Control	6	0.81	0.43
	11 Gy	6	0.79	0.43
	14 Gy	6	0.72	0.43
	17 Gy	6	0.77	0.43
NR1	Control	6	0.03	0.01
	11 Gy	6	0.02	0.01
	14 Gy	6	0.02	0.01
	17 Gy	6	0.02	0.01
NR2A	Control	6	0.03	0.008
	11 Gy	6	0.05	0.008
	14 Gy	6	0.02	0.009
	17 Gy	5	0.04	0.009
NR2B	Control	6	0.03	0.01
	11 Gy	6	0.03	0.01
	14 Gy	5	0.02	0.01
	17 Gy	5	0.02	0.01

## Discussion

A comprehensive behavioral evaluation that examined multiple structure/function relationships was used to determine the effects of whole-brain proton irradiation in adult male rats. Four doses were tested (11 Gy, 14 Gy, 17 Gy, and 20 Gy), and it was determined that the 20 Gy dose was approximately the LD_50_ for this experiment. In a previous study^17^, proton doses ranging from 22–101 Gy delivered at 20 Gy/min were applied to the whole body of rats and no deaths were reported, however the rats were euthanized within 4 days of irradiation. Therefore, a comparison of lethality between that study and ours is not possible given that all the rats exposed to 20 Gy in this study survived for > 4 days after irradiation. Many of the dose-dependent effects found in the present study were observed during weeks 1–5 post-irradiation. In this regard, body weight, open-field, and straight channel all had escalating dose-dependent effects. Interestingly, the cognitive deficits and protein changes in TH and DAT were not dose dependent suggesting a lower threshold for these effects. Compared with controls, there were no differences for unmodified ASR/TSR, rotorod, NOR, or the reversal phase of the MWM, as well as for other physiological markers. The effects are summarized in Table 3.

**Table 3:** Summary of effects of whole brain single fraction conventional proton treatment.

Assessment	Function	Associated primary brain region(s)	Effect—compared with 0 Gy Controls		
			11 Gy	14 Gy	17 Gy
Locomotor activity	Spontaneous locomotion and exploration	Basal ganglia^28^	↓ Wk 2	↓↓ Wk 1–3	↓↓↓ Wk 1–5
Rotorod	Motor coordination	Cerebellum^69^	─	─	─
Novel Object Recognition	Novelty preference	Dorsal hippocampus^27^	─	─	─
Prepulse startle inhibition	Sensory motor gating	Auditory/somatosensory cortex/colliculus^70^	↓	↓	↓
Acoustic and tactile startle	Sensory function	Caudal pontine reticular nucleus^29^	─	─	─
Cincinnati Water Maze (CWM) learning	Egocentric navigation	Striatum^25^	↓↓↓	↓↓	↓
Morris water maze (MWM) learning	Allocentric navigation	Hippocampal/entorhinal cortex^26^	↓	↓	↓↓

The dose dependent increased mortality and decreased body weight after brain irradiation are consistent with the literature on proton treatment^16,33^ as well as with other radiation types^34,35^. The dose dependency observed for body weights was not, however, predictive of cognitive outcomes. Locomotor changes during the first 5 weeks post-irradiation displayed dose-dependent effects and evidence of time-dependent partial recovery. The striatum is an important region in locomotor behavior and has numerous inputs from the motor cortex and other regions^36^. The effect on locomotor behavior was more apparent in the first 3 weeks when all irradiated groups had decreased locomotion compared with the controls, whereas only the 17 Gy was impaired on weeks 4 and 5. No effects on locomotor behavior were found after electron or X-ray irradiation at doses comparable to those used here or higher in some studies^34,35,37^, however decreased locomotion was found after X-ray irradiation when assessed for 6 h overnight three months post-irradiation^38^. Gamma radiation at 10 Gy also decreased locomotion when examined 1–3 days after irradiation but not 4 or 5 days later^39^. Whether the differences are related to the type of radiation, fractionation, or other factors such as the length of locomotor testing (i.e., hours vs. minutes) is unclear. The reductions in locomotion were not generalized to other behaviors. For example, no effects were found for rotorod and unmodified ASR/TSR examined at the same time points that locomotion was examined. Furthermore, there were no differences in swim speed in the MWM. Taken together, these data suggest that the motor pathways were intact even though exploratory behavior was decreased. In support of decreased exploration, the time to reach criterion in NOR was increased in the 17 Gy group compared with controls (*p* < 0.008, Dunnett’s).

In this study, two types of startle stimuli were used, acoustic and tactile^40^. Acoustic stimuli are relayed by the cochlear nucleus whereas tactile stimuli are relayed by the trigeminal nucleus^41^. The cochlear and trigeminal nuclei efferents converge and synapse on the ventral caudal pontine reticular formation that in turn projects to motor neurons resulting in the startle response. After photon irradiation in adult rats (n = 6/group), no differences were noted between groups for ASR while PPI was decreased^38^, however ASR was increased when rats were irradiated as neonates and tested at P210^42^. In the present study, no differences in response were noted when rats were assessed weekly in a lighted chamber for ASR/TSR. These data suggest that the startle response is intact, however when modified by a prepulse signal using a flash of light to inhibit the response^43^, ASR was increased irrespective of prepulse interval; whereas in this context the 17 Gy rats had reduced TSR on week-1. The most consistent ASR increase was with light prepulses in the 11 Gy group, followed by the 17 Gy and then the 14 Gy group. Why the 11 Gy group had more reliable increases in startle is unknown, but suggest that the superior colliculus was affected in this group. The superior colliculus is an important region when light prepulses are used to modify the startle response^44^. A potential issue with an acoustic prepulse is that it depends on the ability of rats to hear above the ambient background of the environment; the light prepulse circumvents this issue. Why acoustic startle was increased in combination with light prepulses is unknown, however several possibilities are worth considering. Firstly, in the unmodified version, the rats were in a lighted chamber, but with the light prepulse the rats were in a dark chamber. Secondly, with prepulse trials there were 20 unmodified trials rather than 50. Statistical examination of the ASR/TSR data where there were only unmodified trials using only the first 20 trials did not change the outcomes. These data suggest that different protocols for ASR and TSR reveal different effects.

Some studies on the effects of irradiation use the NOR or novel object placement tests in rats or mice and report effects on one or the other, although the effects are generally not dose-dependent^19,21,22,34^. No effects of protons were found on NOR in the present study. The perirhinal cortex and hippocampus are important regions for NOR^27,45^. NOR procedures vary and these differences affect outcomes. NOR as first described had a number of variations that included: one or two objects during habituation, a constant object exploration time (20 s), total time in arena held constant, and variable retention intervals (1 min, 1 h, or 24 h)^46^. There are many NOR procedures^47^, including after irradiation (i.e., 2–4 months in the studies by Montay-Gruel et al.^19,23^), and species differences are likely to result in different outcomes across radiation studies. In this experiment, 4 objects were placed in the corners in both phases, and in the retention phase familiar objects were replaced with identical copies of the familiarization objects (to avoid residual odor cues) along with a novel object, rats had to achieve 30 s cumulative object exploration time^27^, and video tracking was used to avoid subjective observer effects. This method produces higher novel object preference compared with 2 object methods, equalizes the time rats spend exploring objects, and is very different from NOR used in previous radiation studies.

While no differences were noted for NOR, there were deficits in egocentric and allocentric learning and memory in proton irradiated rats. These cognitive deficits are consistent with cognitive deficits observed in patients that underwent radiotherapy^1,3,4^. Deficits in the CWM were found for all groups of irradiated rats; here again, the effects were not dose-dependent. Although there was an initial increase in time to reach the platform in the straight channel for the 17 Gy group on trial-1 and for the 14 Gy group on trial-2, no differences remained on the last two trials indicating that all rats were swimming equally before being tested in mazes. Furthermore, no differences in swim speed in the MWM were found further verifying that irradiated rats had no motoric deficiencies. Performance in the CWM is largely mediated by dopamine since dopamine lesions of the striatum with 6-hydroxydopamine induce large learning deficits^25,48,49^. Drugs, pesticides, or metals that target the striatum also produce deficits in the CWM^50–55^. This is the first study to show long-term deficits in the CWM following irradiation. Given that TH, DAT, and DRD1 dopamine markers were decreased in the neostriatum provides converging evidence that the neostriatum is a vulnerable region to proton irradiation and tests of egocentric learning and memory reflect such exposure.

Performance in the MWM is dependent upon the hippocampus^26,56^. Irradiation by various methods increases neurotoxicity in the hippocampus^14,16,17,22^. In some studies, irradiation produced MWM deficits during acquisition^34,37^, reversal^12,20^, or had no effect^38^. In the present study, proton irradiation produced deficits in latency at 11 and 17 Gy in the acquisition phase with all irradiation groups less efficient at locating the platform than controls. It is interesting that no differences were found for the reversal phase of the MWM, but in the shift phase the 17 and 14 Gy rats performed worse than controls. The lack of a reversal effect may be the result of different task requirements for the different phases. For the acquisition phase, rats learn the general task requirements of allocentric learning, namely that the platform is away from the wall and it is located in a stationary position relative to room cues that do not change, and through triangulation of distal objects it allows them to map their surrounding and navigate to the goal. The reversal phase measures cognitive flexibility since rats must forget the previous platform position and locate the goal in a new position, although the position relative to distal cues has not changed. Since the rat knows the task requirements, a reduction in platform size requires more precise navigation. The shift phase requires the rat to forget two previous positions and locate an even smaller platform size in a third position. The interference from two previous positions likely produces a greater retroactive memory burden than reversal and we have found many instances where the shift phase reveals effects not seen on reversal. The differences in outcome across phases provide clear evidence that the task requirements are not the same for each phase and require distinct cognitive processes.

Only the 17 Gy rats were slower at locating the cued platform. The cued phase is a form of egocentric learning but not as difficult as the CWM. Receptor proteins important in hippocampal learning and memory (NMDA receptors) were unaffected by proton irradiation (Table 2). This lack of effect on the glutamate system was observed in other radiation studies as well^20,38^. Therefore, a mechanism for these effects is not yet clear.

Limitations include the use of only male rats. Female rats will be included in future experiments. While we examined several doses, we did not explore the effects of fractionation or varying dose-rates, and we did not vary test order. While test order could be a factor, designing a study with each behavior tested separately is unwieldy and not comparable to humans who are exposed to many learning experiences. Other behavioral tests should be included to examine vulnerabilities to other brain regions. For example, this experiment did not include tests of inhibitory control, working memory, amygdala function, arousal, or anxiety. The present data, however, succeeded in showing that more CNS functions are affected by proton radiation than previously reported. This indicates that a wider range of assessments should be used to avoid missing important effects.

In conclusion, the observations from this study will guide future research using proton irradiation in adult rats using different dose rates and fractionations, and comparisons between proton and X-ray exposures. Given the outcomes, the neostriatum and hippocampus appear to be vulnerable regions from proton irradiation^16,17^. This may result from a number of factors including blood brain barrier integrity, cerebral blood flow changes and other effects^15^. Importantly, these data support findings in humans that proton irradiation has differential effects as a function of brain region and this aspect of radiation effects on brain has not been appropriately appreciated thus far^24^.

## Materials and methods

The protocol for this experiment was approved by the Cincinnati Children’s Research Foundation’s Institutional Animal Care and Use Committee protocol # 2017-0032. All experiments were performed in accordance with relevant guidelines and regulations The subjects were male Sprague–Dawley CD, IGS rats (strain #001, Charles River, Raleigh, NC) weighing 225–250 g (~ 8 weeks of age) upon arrival at the Cincinnati Children’s Research Foundation’s (CCRF) vivarium that is AAALAC International-accredited and pathogen free. Rats were initially pair-housed in polysulfone cages (46 cm × 24 cm × 20 cm) in the Alternative Design (Siloam Spring, AR) MACS Flex-air wall mount system with HEPA filtered air supplied at 30 air changes/h and reverse osmosis filtered, UV purified water provided from a Lixit automated system (SE Lab Group, Napa, CA). Three days prior to proton exposure, rats were transported to the Cincinnati Proton Therapy Center’s vivarium where they were housed in PET plastic cages (43 cm × 34 cm × 20 cm) with an Innocage external bottle system (Innovive, San Diego, CA). Cages contained woodchip bedding, a stainless steel hut for enrichment^57^, and ad libitum access to NIH-07 rat chow (LabDiet #5018, Richmond, IN). Temperature (21 ± 1 °C), humidity (50 ± 10%), and light–dark cycle (14:10 h, lights on at 600 h) were automatically regulated. Rats were returned to the CCRF vivarium 1 week after irradiation and housed as before.

### Proton exposure groups

Rats (n = 10–11/dose) were assigned to a group with the use of a random number table and were individually identified prior to irradiation by ear-punch while anesthetized under 2–4% isoflurane with room air using the SomnoSuite system (Kent Scientific, San Diego, CA). The Cincinnati Children’s/UC Health Proton Therapy Center has a 250 meV cyclotron and ProBeam pencil beam scanning system (Varian Medical Systems, Palo Alto, USA). The beamline delivers proton radiation to a dedicated research room containing a 360° rotating gantry, pencil beam scanning nozzle, laser and X-ray localization systems, and robotic positioner. The research room incorporates dosimetry and treatment equipment identical to that of clinical treatment rooms. The rats were placed in a prone position under the gantry proton beam nozzle to irradiate the entire brain. The radiation field measured 2.5 cm × 3.0 cm and uniformly irradiated the target using transmission exposure with the plateau region of a 245 meV and Bragg peak beneath the rat. Each rat’s brain was localized with the gantry-mounted laser alignment system, such that the inferior edge of the field and the inferior/posterior edge of the rat brain were aligned. Rats received physical doses of 0, 11, 14, 17, or 20 Gy proton radiation in a single fraction at 1 Gy/s; each treatment plan was quality assured with tolerances of 2% of the absolute dose and 5% of the dose rate. Prior to radiation, field flatness was quality assured using gafchromic film. Treatment doses were verified using an NIST-traceable ionization chamber. Dose calculations for ionization chambers use the dose-to-water formulism as reported in the IAEA TRS 398. After exposure, rats were allowed to recover from anesthesia prior to return to the vivarium. Rats were given Napa Nectar pouches (Systems Engineering Lab Group, Napa, CA) in the cages for 6 weeks as a secondary source of hydration. Body weights were recorded at the time of exposure and weekly thereafter.

### Behavior

Rats were tested beginning 1 week after irradiation and weekly for 5 weeks post-irradiation days (PID)—for spontaneous locomotor activity in an automated open-field, ASR and TSR, rotorod, and light prepulse inhibition of ASR or TSR. Starting 6 weeks after irradiation, rats received cognitive assessment for NOR, followed successively by straight channel swimming, CWM, and MWM. Non-water maze equipment was cleaned between rats with EPA approved Process NPD solution (Steris, Mentor, OH). Behavioral testing was conducted during the light cycle in the Animal Behavioral Core of CCRF. Personnel performing testing were blind to group membership. Brain tissues were collected at the end of behavioral testing.

#### Locomotor activity

On the first test day of each week (PID 7, 14, 21, 28, and 35), rats were placed in 41 cm × 41 cm PAS activity chambers (San Diego Instruments (SDI), San Diego, CA) for 60 min as described^58^. Data were recorded in 5 min intervals for 60 min. Rats were returned to their home-cage for at least 1 h prior to ASR/TSR testing.

#### Acoustic and tactile startle

On the same day as locomotor activity, ASR and TSR were measured in SR-LAB test chambers (SDI) as described and equipped for rats^59^. Rats were placed in acrylic cylindrical holders (SDI large enclosure) mounted on a platform with four legs with a piezoelectric accelerometer attached to the underside; the platforms were located inside sound-attenuating chambers with a house light and fan turned on. Prior to each session, there was a 5 min adaptation period to the holders with no stimuli presented. For ASR the pulse was a 20 ms, 120 dB SPL mixed frequency white noise burst (rise time 1.5 ms). For TSR the pulse was a 20 ms, 60 psi air-puff directed at the dorsum of the rat through a plastic tube. The recording window lasted 100 ms from onset of the pulse. Maximum startle amplitude (V_max_) was measured in mV. Any detected movement prior to trial onset was subtracted from V_max_. Rats were given 5 acoustic (120 dB) trials alternating with 5 tactile (60 psi) trials for a total of 100 trials (50 of each type). Vmax is reported since the average response (V_avg_) is highly correlated with V_max_ (r > 0.95) see Refs.^60,61^.

#### Rotorod

Rats were tested on the Rotor Rod (SDI) apparatus the day after locomotor and ASR/TSR testing each week (PID 8, 15, 22, 29, and 36). Rats were placed on a 7 cm diameter rod with an initial rotation rate of 4 rpm. Once the test started there was a stepwise acceleration to 40 rpm over the next 6 min. The trial ended at 6 min or when the rat fell to a padded surface. Rats were tested for 4 trials/week with an intertrial interval on each day of 15 min. Latency to fall was analyzed.

#### Bright light prepulse startle

Approximately 1 h after rotorod, rats were tested for ASR and TSR, using the same conditions as above, with two changes: (1) house lights were off and (2) before the acoustic or the tactile pulse there was a light (~ 1110 lx) prepulse presented at interstimulus intervals of 30, 70, 100, or 400 ms or with no prepulse. The light prepulse was from an LED array of high intensity lights (SDI) that provided approximately 1110 ± 17 lx (mean ± SEM) measured at the level of the animal holder with a light meter (Extech Easyview 33, Boston, MA). Light prepulses do not elicit a startle response by themselves. A 10 × 10 Latin square of each prepulse/pulse trial type was repeated until 20 trials of each were obtained. V_max_ was the dependent measure.

### Cognitive assessment

The order of the cognitive tests was to assess NOR before water mazes.

#### Novel object recognition (NOR)

Approximately 6 weeks post-irradiation (~ PID 42), NOR was tested in a 40 cm × 40 cm × 40 cm high acrylic AnyBox apparatus (Stoelting Co., Wood Dale, IL) with a camera mounted above attached to a computer that ran ANY-maze to track movement (Stoelting Co.). The test has two phases: familiarization and retention. Familiarization: Rats were placed in the test arena with four identical objects, one in each corner. Object exploration was scored when the rat was within 1 cm of an object and facing it. To ensure that rats had comparable object exploration, rats had to have 30 s of cumulative exploration of the objects within 10 min^27^. Only 1 rat (14 Gy) failed to reach criterion and was not included in the analysis. Retention testing began 1 h later and had three identical copies of the original objects plus one new object in each corner. Rats again remained in the arena until 30 s of cumulative object exploration occurred up to a limit of 10 min. The percent time spent with the novel object (novel object time in 30 s) was the dependent variable. Chance exploration for 4 objects is 25%.

#### Straight channel

Following NOR, rats were tested in a straight swimming channel (~ PID 43). Rats were given four timed consecutive trials in a 244 cm long × 15 cm wide × 50 cm high straight channel filled halfway with water with a submerged (~ 1–2 cm) escape platform at one end. Each trial started with the rat placed in the channel facing away from the platform at the opposite end. The straight channel provides rats with experience finding the escape and recording latencies to reach the goal ensures comparable swimming ability prior to the start of maze testing. This procedure is a necessary step for rats to learn in the CWM. Latency to escape was analyzed.

#### Cincinnati water maze

The day following straight channel, rats were assessed in the CWM for 18 days (~ PID 44-62). The CWM consists of 10 T-shaped cul-de-sacs branching from a channel extending from the start to the goal^55^. The maze is 51 cm tall and filled halfway with water (21 ± 1 °C). To exclude distal cues, testing was conducted in the dark under infrared light. An infrared sensitive camera was mounted above the maze and connected to a monitor in an adjacent room where the experimenter scored latency to escape and errors. Prior to testing, rats were acclimated to the dark for at least 5 min. Rats were given 2 trials/day with a 5 min limit/trial for 18 days. If rats found the platform in under 5 min on trial-1, they were given trial-2 within 30–60 s. If they did not find the goal within 5 min on trial-1, they were removed from the maze, not guided to the exit and rested for 5-min before trial-2. An error was counted if the head and forelimbs entered into the stem of any dead-end cul-de-sac or into the crossing-arm of a “T”. After each day of testing, rats were dried by placement in a cage with thick towels for 5 min to wick away most of the water before being returned to their home cage. The latency to find the submerged platform and errors were the dependent variables. Rats that did not reach the goal in 5 min were given an error correction that was the number of errors from the rat with the greatest number of errors to compensate for rats that stopped searching and made few errors but took 5 min.

#### Morris water maze

Lastly, rats were tested in the MWM for 23 days (~ PID 63-85). The maze is a circular black pool 244 cm in diameter and 51 cm deep with a conical bottom, filled with room temperature water (21 ± 1 °C) to a depth of 25 cm with a black platform submerged 2 cm below the surface in one quadrant of the pool. The platform was camouflaged by virtue of being black against a black background. The walls surrounding the maze have posters and geometric shapes mounted so they are visible from water level. Performance was tracked with AnyMaze (Stoelting, Inc., Wood Dale, IL). Rats were tested in 4 phases: acquisition, reversal, shift, and cued-random^62–64^. For acquisition, reversal, and shift there were 4 platform trials/day for 6 days and 2 probe trials (no platform), one at the beginning of day-3 and one 24 h after the last trial on day-6. The time limit for probe trials was 45 s. For the probe trials, dependent measures were average distance from the former platform position^65^ and number of site crossovers. The time limit on learning trials was 2 min/trial and the intertrial interval was 5 s spent on the platform plus the time to run other rats in rotation in each test cohort (3–10 min). For acquisition the platform was 10 cm diameter and located in the southwest quadrant (north was defined as the position furthest from the experimenter), for reversal the platform was 7 cm diameter and located in the northeast quadrant, and for shift the platform was 5 cm diameter and located in the northwest quadrant. The reduced platform sizes require rats to demonstrate increased spatial accuracy to find the goal^62,64^. For the learning trials, latency, path efficiency, and swim speed were analyzed. Path efficiency is calculated as a straight line from the start position to the goal divided by the path length the rat took to reach the goal. Similar to the platform phases the cued-random phase had 4 trials/day, but for only 2 days, and the submerged platform (10 cm) was marked with a yellow ball mounted on a stainless-steel rod that protruded 10 cm above the water. Distal cues were blocked by black curtains that were drawn closed around the pool. The platform and start positions were moved on each trial so that spatial cues could not be used to triangulate the location of the platform^62^. The dependent measure for cued-random was latency to the platform since video tracking with the curtains closed was not possible. These phases test explicit/spatial/allocentric learning and reference memory (acquisition), cognitive flexibility (reversal), and retroactive interference (shift). The cued-random phase is a form of egocentric learning that controls for vision, ability to use proximal cues, motivation to escape, and swimming ability.

### Brain tissue collection

Approximately 2 weeks after the completion of behavior (~ PID 99), rats were rapidly decapitated without anesthesia and brains collected. Neostriatum and hippocampus were dissected bilaterally over ice as described after cutting the brain in a brain block in 2 mm coronal slices^66^. Each region was placed in conical tubes, on dry ice, and stored at − 80 °C until assayed.

### Western blot analysis

Western blots were used to analyze dopamine, glutamate, astrocytic, and microglial biomarkers in the neostriatum and hippocampus as described^58^. They included tyrosine hydroxylase (TH), dopamine transporter (DAT), and dopamine receptor D1 (DRD1). Analyses were performed on six rats from each of the behaviorally tested groups; actin was used as reference. Frozen tissues were homogenized in immuno-precipitation assay buffer [25 mM Tris, 150 mM NaCl, 0.5% sodium deoxychlorate, and 1% Triton X-100 adjusted to 7.2 pH with protease inhibitor (Pierce Biotechnology, Rockford, IL)]. Protein was quantified using the BCA Protein Assay Kit (Pierce Biotechnology, Rockford, IL) and diluted to 3 µg/µL. Western blots were performed using LI-COR Odyssey (LI-COR Biosciences, Lincoln, NE) procedures. Briefly, 25 µL of sample was mixed with Laemmli buffer (Sigma, USA) and loaded on a 12% gel (Bio-Rad Laboratories, Hercules, CA) and run at 200 V for 35 min in running buffer (25 mM Tris, 192 mM glycine, 0.1% SDS). The gel was transferred to Immobilon-FL transfer membrane (Millipore, USA) in 1× rapid transfer buffer (AMRESCO, Solon, OH) at 40 V for 1.5 h. Membranes were soaked in Odyssey phosphate buffered saline blocking buffer for 1 h and incubated overnight at 4 °C with primary antibody in blocking buffer with 0.2% Tween 20. Membranes were incubated with secondary antibody in blocking buffer with 0.2% Tween 20 and 0.01% SDS for 1 h at room temperature. Western blot analyses in the neostriatum were conducted with the following antibodies and dilutions: rabbit anti-tyrosine hydroxylase (ab112, Abcam, Cambridge, MA) at 1:1,000 with Odyssey IRDye 800 secondary body used at a 1:2000 dilution; rabbit anti-dopamine transporter (ab184451, Abcam, Cambridge, MA) at 1:2000 with Odyssey IRDye 800 secondary antibody at 1:20,000 dilution; rabbit anti-dopamine receptor D1 (ab40653, Abcam, Cambridge, MA) at 1:1000 with Odyssey IRDye 800 secondary antibody at 1:3000 dilution; chicken anti-rabbit glial fibrillary acidic protein (ab4674, Abcam, Cambridge, MA) at 1:2000 with Alexa Fluor 647 secondary antibody at 1:4000 dilution; mouse anti-Ki67 (SC-23900, Santa Cruz Biotechnology, Dallas, TX) at 1:20 with Odyssey IRDye 800 secondary antibody at 1:750 dilution; and mouse anti-allograft inflammatory factor 1 (SC-32725, Santa Cruz Biotechnology, Dallas, TX) at 1:250 with Odyssey IRDye 800 secondary antibody at 1:1000 dilution. Western blot analyses in the hippocampus were performed for subunits of the NMDA receptors, GFAP, and Ki67^67^ with the following antibodies and dilutions: rabbit anti-NMDA receptor 1 (ab109182, AbCam, Cambridge, MA) at 1:4000 with Odyssey IRDye 800 secondary antibody at 1:3000 dilution; rabbit anti-NMDA receptor 2A (ab124913, Abcam, Cambridge, MA) at 1:9000 with Odyssey IRDye 800 secondary antibody at 1:20,000 dilution; rabbit anti-NMDA receptor 2B (ab81271, Abcam, Cambridge, MA) at 1:5000 with Odyssey IRDye 800 secondary antibody at 1:20,000 dilution. GFAP and Ki67 dilutions were the same as performed in the neostriatum. Mouse anti-actin (Ab3280, AbCam, Cambridge, MA) at 1:2000 with Odyssey IRDye 680 at a 1:15,000 secondary antibody was used as a loading control. Relative protein levels were quantified using the LI-COR Odyssey scanner and Image Studio software for fluorescent intensity of each sample normalized to actin.

### Statistical procedures

Data were analyzed using mixed linear ANOVA models (SAS Proc Mixed, SAS Institute 9.4 TS, Cary, NC). Proc Mixed accommodates only 1 repeated measure over time, therefore tests that were repeated weekly and had a trial or time component were analyzed separately for each week. Repeated measure factors were fit to either the autoregressive moving average or the autoregressive covariance structure depending upon best fit determined by the corrected Akaike Information Criterion. Repeated measures were week (body weights and ASR/TSR), day (CWM and MWM), time (locomotion), or trial (ASR/TSR with prepulse, rotorod, straight channel). The estimation method for the covariance parameters was by the restricted maximum likelihood method, with the exception of the CWM data where minimum variance quadratic unbiased estimation was used. Kenward-Roger first order adjusted degrees of freedom were estimated for Type III ANOVAs and these can be fractional^68^. Only deficits in learning and memory were predicted and Dunnett’s tests were used to compare each irradiated group with controls; for these only *p* values are provided. Interactions were tested with mixed linear ANOVAs. Significant interactions were further analyzed using slice-effect ANOVAs and Dunnett’s tests. Significance was set at *p* ≤ 0.05. Data are presented as least square (LS) mean ± SEM. For preplanned comparisons *p*-values are given since Dunnett’s test does not require an F statistic. Unless stated in the figure legends, all groups had n = 10 with the exception of the 20 Gy group that had n = 11.

## Supplementary information

Supplementary Information.
