# Supplementary material for: Whole brain proton irradiation in adult Sprague Dawley rats produces dose dependent and non-dependent cognitive, behavioral, and dopaminergic effects
Source: Sci Rep. 2020 Dec 9;10:21584. doi: 10.1038/s41598-020-78128-1 (PMC7726106; doi:10.1038/s41598-020-78128-1)
Supplement: Supplementary file 1 — Supplementary Information. [file 41598_2020_78128_MOESM1_ESM.docx]

**Whole brain proton irradiation in adult Sprague Dawley rats produces dose dependent and non-dependent cognitive, behavioral, and dopaminergic effects**

Michael T. Williams*^1,2,5^, Chiho Sugimoto^1^, Samantha L. Regan^1,2^, Emily M. Pitzer^1,2^, Adam L. Fritz^1^, Anthony E. Mascia^3,5^, Mathieu Sertorio^2,4,5^, Ralph E. Vatner^3,5^, John P. Perentesis^2,4,5^, Charles V. Vorhees^1,2,5^

*^1^Division of Neurology, Cincinnati Children’s Research Foundation, and ^2^Department of Pediatrics, University of Cincinnati College of Medicine, Cincinnati, OH, USA 45229*

*^3^Department of Radiation Oncology, University of Cincinnati College of Medicine, Cincinnati, OH, USA 45267*

*^4^Division of Oncology, Cincinnati Children’s Research Foundation, Cincinnati, OH, USA 45229*

*^5^Cincinnati Children’s/University of Cincinnati Proton Therapy and Research Center, Cincinnati, OH, USA 45229*

*^1^Division of Neurology, Cincinnati Children’s Research Foundation, and ^2^Department of Pediatrics, University of Cincinnati College of Medicine, Cincinnati, OH, USA 45229*

**Supplementary Figures and Figure Legends**

**Figure S1.** Original full western blot image of TH (a), DAT (b), and DRD1 (c). These data are unadulterated images of the western blots in the paper. The blots represented in the paper are these images cropped in the middle as to not show duplicates. White text was added above each lane to indicate the control and exposure groups.
